# Liver regeneration: immunohistochemichal study of intrinsic hepatic innervation after partial hepatectomy in rats

**DOI:** 10.1186/s12876-014-0202-1

**Published:** 2014-11-25

**Authors:** Apostolos N Kandilis, John Koskinas, Ioannis Vlachos, Spyridon Skaltsas, Despina Karandrea, Petros Karakitsos, Alkistis Pantopoulou, Marina Palaiologou, Nikolaos Nikiteas, Dina G Tiniakos, Despina N Perrea

**Affiliations:** Second Propedeutic Department of Surgery, Medical School, University of Athens, General Hospital “Laiko”, Athens, Greece; Second Department of Medicine, Medical School, University of Athens, “Hippokration” Hospital, Athens, Greece; Laboratory for Experimental Surgery and Surgical Research “N. S. Christeas”, Medical School, University of Athens, Athens, Greece; Laboratory of Histology and Embryology, Medical School, National and Kapodistrian University of Athens, Athens, Greece; Department of Pathology, Aretaieion Hospital, Athens, Greece; Department of Cytopathology, University General Hospital “ATTIKON”, School of Medicine, National and Kapodistrian University of Athens, Athens, Greece; Institute of Cellular Medicine, Faculty of Medical Sciences, Newcastle University, William Leech Bldg, 4th Floor, Room M4.143, Framlington Place, Newcastle upon Tyne, NE2 4HH UK

## Abstract

**Background:**

We examined the intrinsic hepatic innervation after partial hepatectomy (PH) in rats and the presence and pattern of neural sprouting in regenerating liver.

**Methods:**

Male Wistar rats (age 9–13 weeks-w, weight 204-356 g), were submitted to two-thirds PH. Rats were sacrificed at postoperative days (d) 1, 3, 5, 7, at 2 and 4 w, and at 3 and 6 months (m) (6–7 animals/group, control group n = 4). Immunohistochemistry for the pan-neural marker protein gene product 9.5 (PGP9.5) and growth-associated protein 43 (GAP-43), a marker of regenerating nerve axons, was performed on tissue sections from the R1 lobe of the regenerating liver. Portal tracts (PTs) with immunoreactive fibers were counted in each section and computer-assisted morphometric analysis (Image Pro Plus) was used to measure nerve fiber density (number of immuno-positive nerve fibers/mm^2^ (40x)).

**Results:**

Immunoreactivity for PGP9.5 was positive in all groups. The number of PGP9.5 (+) nerve fibers decreased from 0.32 +/− 0.12 (control group) to 0.18 +/− 0.09 (1d post-PH group), and gradually increased reaching pre-PH levels at 6 m (0.3 +/− 0.01). In contrast, immunoreactivity for GAP-43 was observed at 5d post-PH, and GAP-43 (+) PTs percentage increased thereafter with a peak at 3 m post-PH. GAP-43 (+) nerve fiber density increased gradually from 5d (0.05 +/− 0.06) with a peak at 3 m post-PH (0.21 +/− 0.027). At 6 m post-PH, immunoreactivity for GAP-43 was not detectable.

**Conclusions:**

Following PH in rats: 1) nerve fiber density in portal tracts decreases temporarily, and 2) neural sprouting in the regenerating liver lobes starts at 5d, reaches peak levels at 3 m and disappears at 6 m post-PH, indicating that the increase in hepatic mass after PH provides an adequate stimulus for the sprouting process.

## Background

The extraordinary ability of the liver to regenerate following injury or resection is a property that was recognized by ancient Greeks in the well-known myth of Prometheus and the less known myth of Tityus [1]. Liver regeneration is a very complex process involving the activation and interaction of multiple cytokines and growth factors that regulate cell growth and proliferation. During the regenerative process after partial hepatectomy (PH), liver cells continue to function, while undergo mitosis in order to re-establish the organ’s mass. In the rat, restoration of hepatic mass is completed in 5–7 days following PH, whereas liver architecture in terms of sinusoidal ultrastructure is restored in 10–14 days [2-6]. On the other hand, little is known about the nerves in the regenerating liver. Ungvary et al. were the first to study the effect of PH on the monoaminergic nerves of the liver [7]. Pietroletti et al. studied hepatic innervation in a rat model after PH with the use of immmunohistochemistry [8], and Carobi examined the possibility of neural sprouting in the regenerating rat liver following PH [9].

Hepatic re-innervation after experimental orthotopic liver transplantation (OLT) has been studied in the past using immunohistochemistry with antibodies to protein gene product 9.5 (PGP 9.5) and growth-associated protein-43 (GAP-43) in rat models [10,11]. PGP9.5 belongs to the ubiquitin carboxy-terminal hydrolases [12]. It is expressed in neurons and neuroendocrine cells of vertebrates, and is present within the axoplasm of both peripheral and central nerve fibers, thus rendering it an excellent marker for nerve axons [13]. An important drawback of PGP9.5 is the lack of discrimination between normal and regenerating axons [11]. GAP-43 is a protein exclusively expressed in the nervous system. Its expression is related to axonal growth during neuronal development and regeneration and, therefore, GAP-43 is a useful marker for developing or regenerating nerve axons [14-16].

The present study was conducted to examine alterations of the intrinsic hepatic innervation at several time points following PH in rats, using the neuronal markers PGP9.5 and GAP-43. In addition, the possible role of the increase in hepatic mass after PH as an adequate stimulus for neural sprouting was evaluated.

## Methods

Fifty six male Wistar rats, with a mean weight of 283 g, were purchased by the National Centre of Scientific Research “DEMOKRITOS” (Athens, Greece). The animals had free access to water and food, and 12 h before surgery they were deprived only of food. Four/56 animals were randomly chosen to consist the control group and were not submitted to an operation, while the remaining 52 were submitted to two-thirds partial hepatectomy (PH), according to Higgins and Anderson [17], under ether anesthesia. Following PH, rats were sacrificed at postoperative days (PODs) 1, 3, 5, 7, at 2 and 4 weeks, and at 3, and 6 months post-PH. Each group consisted of 6 or 7 animals. The liver was then removed en block and crosscut, 3-mm-thick tissue specimens were obtained from the anterior sub-lobe (R1) of the rat liver right lobe [18].

The study was approved by the Ethics Committee of the National and Kapodistrian University of Athens and was carried out according to the strict regulations concerning animal care set by this committee.

### Immunohistochemistry

Tissue blocks consisting of two or three crosscut specimens from the R1 lobe were fixed for 24 h in 10% neutral formalin and then were routinely paraffin-embedded. Five-μm-thick serial sections were cut from each block and were mounted onto poly-L lysine-coated slides. One section/case was stained with haematoxylin and eosin stain for conventional histological evaluation. Immunochemistry was performed manually using the Novo Castra Novolink Polymer Detection Kit, according to the manufacturer’s instructions. After deparaffinization and rehydration of the sections in a series of graded ethanols, endogenous peroxidase was quenched with 1% H2O2 in methanol for 30 minutes (min). After three 4-min treatments in 0.001 M citrate buffer (pH = 6) in a microwave oven (800 W) for antigen unmasking, sections were incubated overnight in 4°C with the primary antibodies specific for PGP9.5 (Novocastra Laboratories, UK, mouse CLONE 1DA1, diluted 1:100) and GAP-43 (Zymed Laboratories, USA, mouse CLONE 7B10, diluted 1:150), followed by incubation with the secondary antibodies coupled with polymer-horseradish peroxidase (Novolink Polymer) for 25 min at room temperature. All steps were carried out in a 0.25 M phosphate buffer saline (PBS; pH = 7.4) at 25°C. After being rinsed with tap water, sections were counterstained with hematoxylin for 15 sec, dehydrated in graded ethanols, cleared in xylene, mounted with DPX (BDH, England), and coverslipped. A negative control, in which the primary antibody was omitted, was used in every case. Sections of the central nervous system of a rat embryo were used as positive controls for both neuronal markers, as previously reported [11].

### Computer-assisted morphometric analysis of immunoreactive nerve fibers

Morphometric variables were determined by image analysis, using an IBM computer (IBM Computer, Inc.; Armonk, NY). Images were captured with a Leica DM LB microscope coupled to a Sony digital camera (DFW-X700) and digitalized with Path-Sight version 4.3 (Medical Solutions plc.; Nottingham, UK).

We examined a mean of 7 portal tracts (PTs) per section (range 5–14). Each section was obtained from the R1 lobe of the liver rat, 0.5-1 cm from the hepatic hilus. Hot spot areas, i.e. PTs with positive immunostaining were further analyzed using Image Pro Plus (Media Cybernetics, Inc.; Silver Spring, MD). The size of the PTs was not always small enough to fit entirely into one single image (photograph) by the digital camera; therefore serial images were taken, each of them consisting of a variable number of immunostained nerve fibers. The computerized system measured the number of nerve fibers per mm^2^ in the hot spot areas of 40x objective/section/region [19]*.*

### Statistical analysis

Data are expressed as mean ±1 standard deviation (S.D.). The normality of the distributions was assessed with Kolmogorov-Smirnov test and graphical methods. Comparisons between more than two groups were performed with Analysis of Variance (ANOVA). In cases of multiple hypothesis testing, Benjamini & Hochberg’s False Discovery Rate (FDR) was utilized in order to assess between-group differences, as well as to control family-wise error to <0.05. The GAP-43 and PGP9.5 models were implemented using cubic B-Splines. All tests were two-sided. Differences were considered as statistically significant if the null hypothesis could be rejected with >95% confidence (p < 0.05).

## Results

### Immunoreactivity for PGP9.5

**Figure 1:**
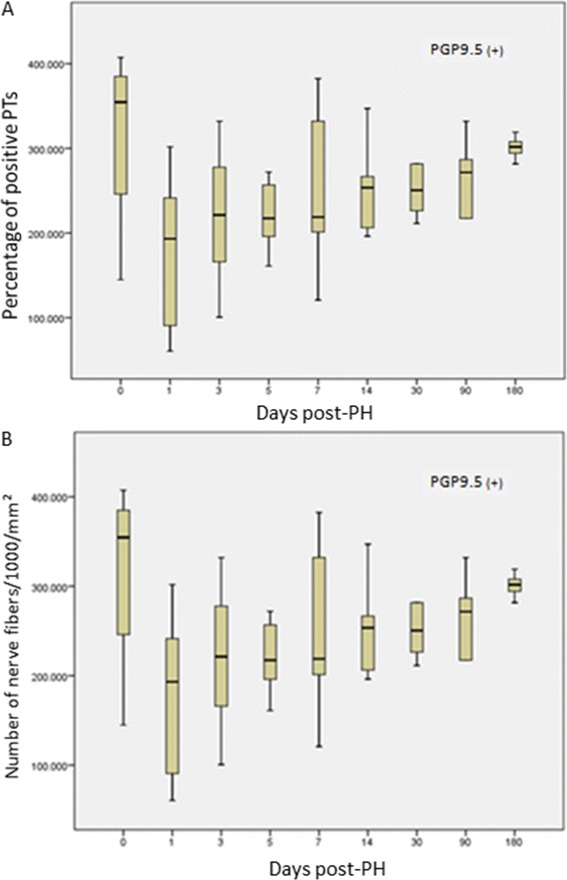
**Change in immunoreactivity for PGP9.5 following PH** ***.*** **A**. Fluctuation of percentage of PGP 9.5 (+) PTs, **B**. Fluctuation of density of PGP 9.5 (+) nerve fibers/mm^2^.

**Figure 2:**
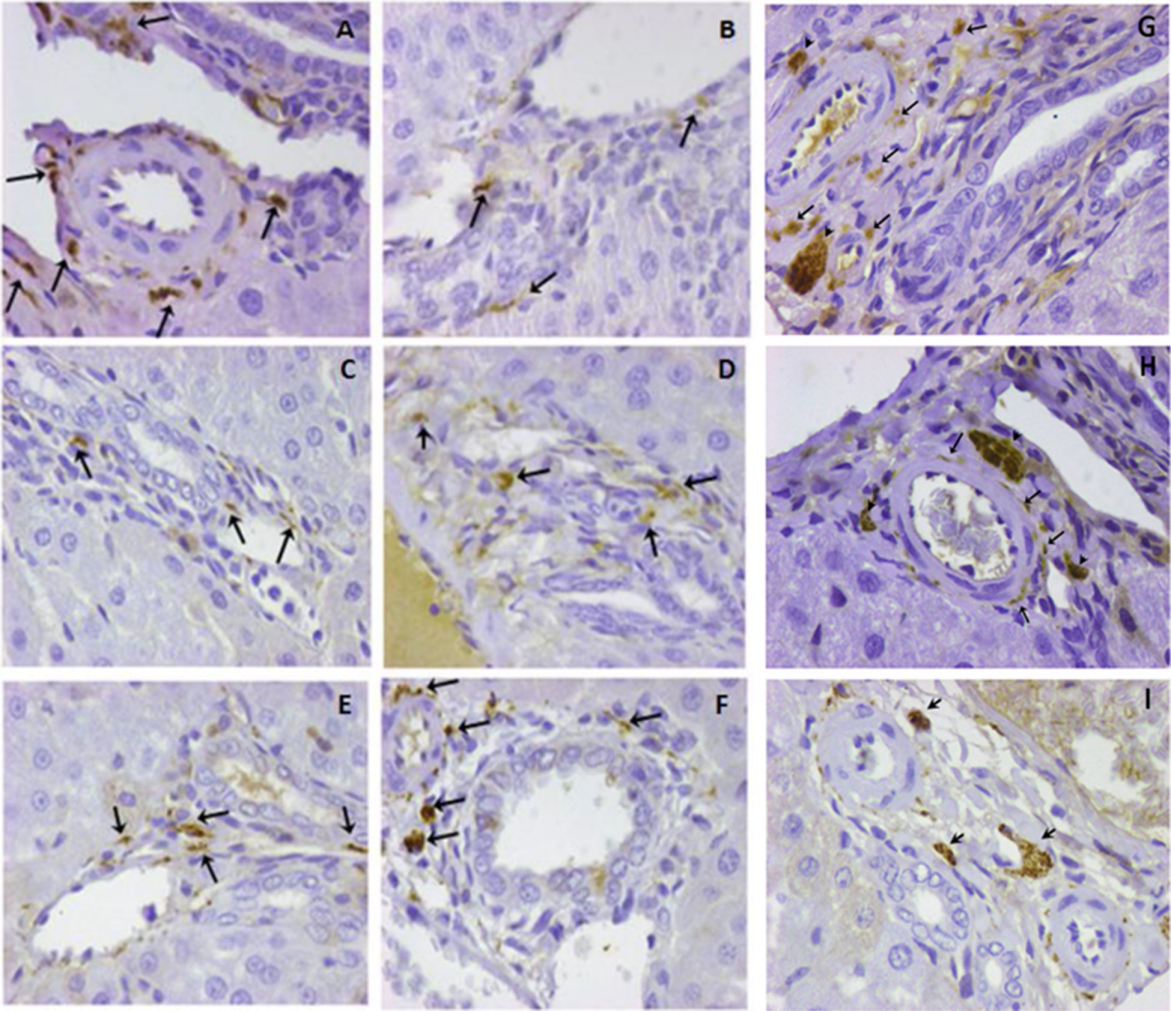
**Immunoreactivity for PGP9.5 (40x). A**. Control group, **Β**. POD 1, **C**. POD 3, **D**. POD 5, **E**. 1 week post-PH, **F**. 2 weeks post-PH, **G**. 1 month post-PH, **H**. 3 months post-PH, **I**. 6 months post-PH. Increased density of nerve fibers at the control group, in the form of dots and small nerve bundles (arrows), as well as at 1, 3 and 6 months post-PH, in the form of dots (arrows) and nerve bundles (arrow heads).

PGP9.5-positive nerve fibers were detected during the entire study period, with alterations in their density (number of nerve fibers/mm^2^) and number of positive portal tracts (PTs) (Figure 1A). In all instances, the immunohistochemical reaction, when positive, was localised exclusively in PTs (Figure 2). At POD 1, nerve fiber density was substantially decreased at 0.180 (+/− 0.091) in comparison to the control group (0.315 +/− 0.116) (p = 0.0507). Thereafter, density gradually increased to 0.220 (+/−0.084) at POD 3, 0.245 (+/− 0.995) at 1 week, 0.266 (+/− 0.044) at 3 months, and, lastly, reached the pre-hepatectomy level at 6 months post-PH (0.30 +/− 0.013) (Figure 1B).

Furthermore, at POD 1, the percentage of PGP9.5-positive PTs was decreased to 46.42% (44.67 +/− 12.98) from 58.62% (59.00 +/− 7.26) in the control group (p > 0.05) and gradually increased to 56.33% (56.14 +/− 7.358) at 6 months post-PH.

### Immunoreactivity for GAP-43

**Figure 3:**
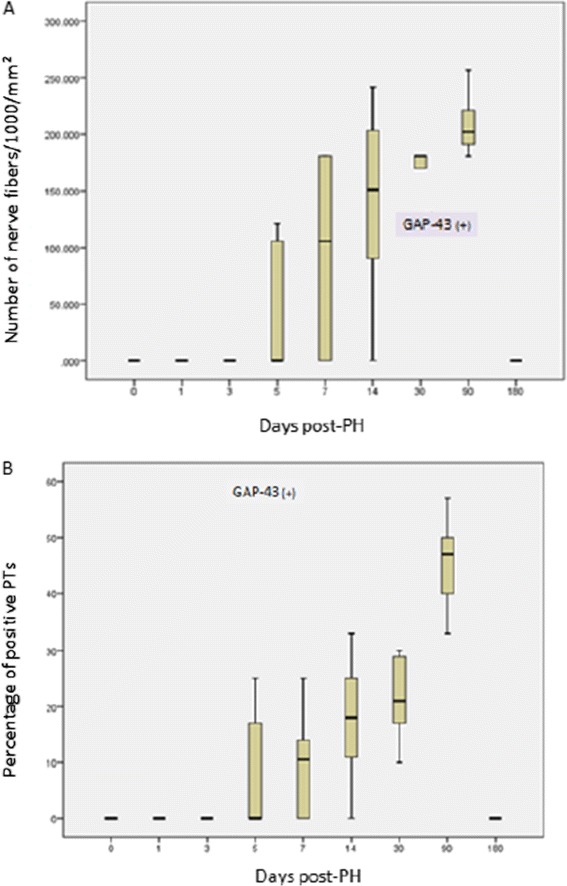
**Change in immunoreactivity for GAP-43 following PH. A**. Fluctuation of density of GAP-43 (+) nerve fibers/mm^2^, **Β**. Fluctuation of percentage of GAP-43 (+) PTs.

**Figure 4:**
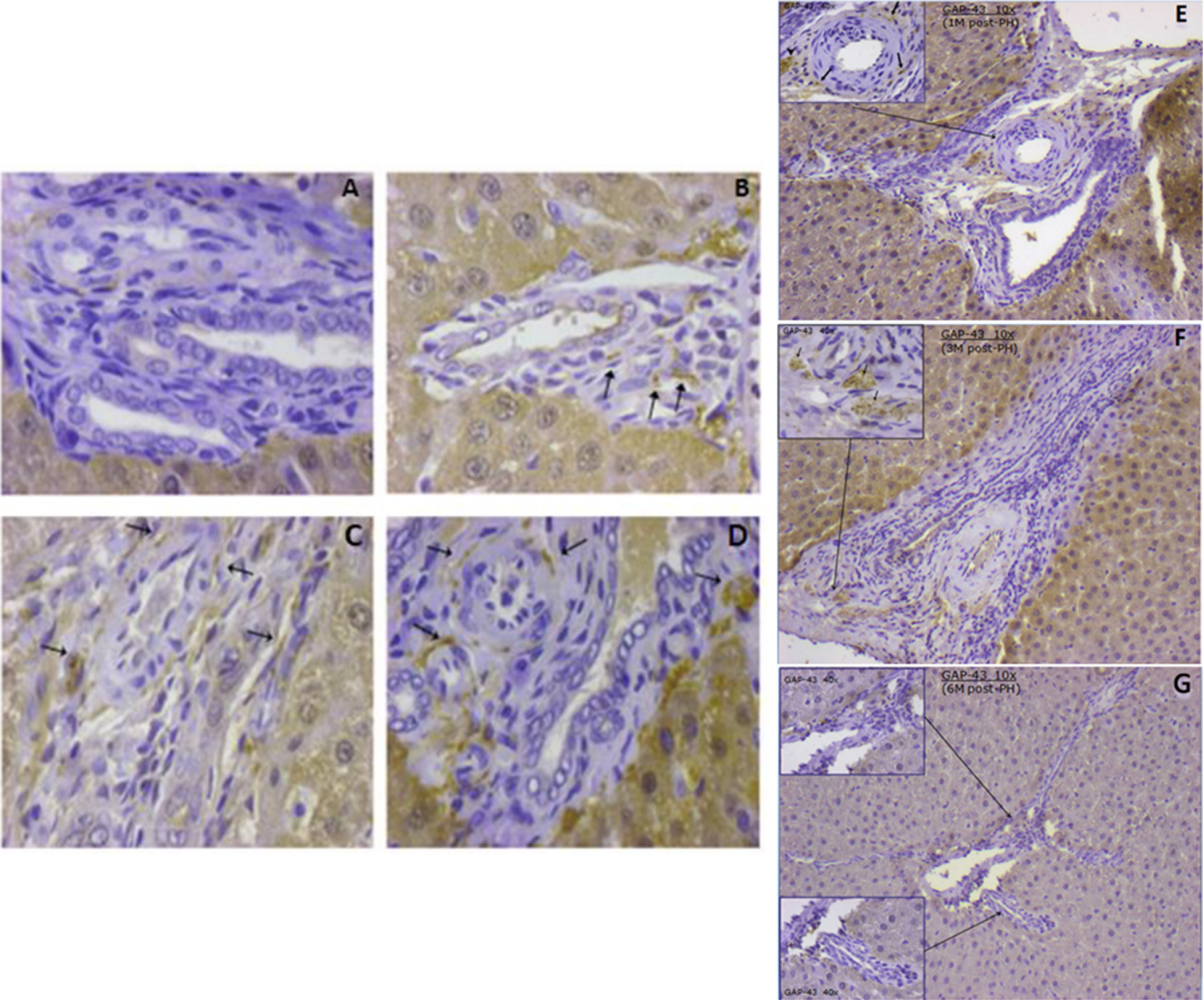
**Immunoreactivity for GAP-43. A**. POD 3 (40x), **B**. POD 5 (40x), **C**. 1 week post-PH (40x), **D**. 2 weeks post-PH (40x), **E**. 1 month post-PH, **F**. 3 months post-PH, **G**. 6 months post-PH. No immuno-positive nerves at POD 3 and expression of GAP-43-positive fibers at POD 5 with increased density at 1 and 2 weeks and 1 and 3 months post-PH, in the form of dots (arrows) and nerve bundles (arrows at F).

GAP-43 immunoreactivity was absent in the control group (0/4) and at POD 1 and 3. GAP-43 positive nerve fibers were firstly identified at POD 5 in PTs of some specimens (3/7), whereas no immunoreactivity was evident in the control group (0/4) and at PODs 1 (0/6) and 3 (0/7). Thereafter, GAP-43 was expressed in all specimens at 1 (6/6) and 3 (6/6) months post-PH. At 6 months post- PH, no GAP-43-positive nerves (0/7) were detected (Figure 3). Similarly to PGP9.5 immunostaining results, GAP-43-specific immunopositivity was localised exclusively at PTs (Figure 4). At POD 5, when GAP-43-immunopositivity was first detected, the density of GAP-43-positive fibers was 0.047 (+/− 0.060). It gradually increased to 0.095 (+/− 0.081) at 1 week, 0.140 (+/− 0.087) at 2 weeks, 0.169 (+/− 0.024) at 1 month, to reach a peak at 3 months (0.210 +/− 0.027) post-PH (Figure 3Α). The difference in density between the groups at POD 5 and 1 week post-PH was not statistically significant (p > 0.05), whereas differences between POD 5 and 2 weeks, 1 month and 3 months post-PH were statistically significant at a level of p < 0.01, p < 0.001, and p < 0.0001, respectively. No intraparenchymal GAP-43-positive fibers were observed in either the control or the experimental group.

At POD 5, the mean percentage of GAP-43-positive PTs was 8.43% (+/− 11.22), then increased steadily to 10.0% (+/− 9.40) at 1 week, 17.57% (+/− 11,15) at 2 weeks, 21.33% (+/− 7.53) at 1 month to reach the highest level at 3 months (45.67% +/− 8.50) post-PH (Figure 3Β).

## Discussion

We have shown that during liver regeneration after PH, the number of PGP9.5-positive nerve fibers was decreased at POD 1 and subsequently gradually recovered reaching the levels of the control group at 6 months. Furthermore, PGP9.5-positive axons were present exclusively in PTs of every specimen at all time points after PH, whereas the expression of GAP-43 became positive in PTs at POD 5, increased to a peak at 3 months, and disappeared at 6 months post-PH.

The exclusive co-expression of PGP9.5- and GAP-43-positive nerve fibers in portal tracts is related to the specific nerve distribution in the rat liver. Intrahepatic nerve distribution is highly species-dependant and the most prominent feature in the rat is the lack of intra-lobular innervation and mere presence of nerve fibers in PTs [20]. In contrast, human liver shows high nerve density in the lobules, while nerve fibers are also detected in PTs [21].

The transient reduction of PGP9.5 positive axons early after PH is in keeping with the results of Ungvary et al. and can be attributed to the prevalence of liver cell proliferation in order to restore the organ’s mass, proceeding in a faster manner than the innervation of the regenerating liver [7]. After examining the monoaminergic nerve fibers, Ungvary et al. found no innervation at the periphery of the regenerating lobules at POD 1, and restoration of nerve density at 6 weeks post-PH. Such an alteration was not encountered at the hepatic hilus, where, on the contrary, increased innervation was observed at POD 7 [7]. Hyperinnervation was also noted by Pietroletti et al. in their immunohistochemical study of the neuronal markers neuron specific enolase, neurofilaments and protein S-100 in rats after PH [8]. In addition to the importance of the dual blood supply for the optimal regenerative response, they detected more intense immunoreactivity and more nerve fibers in the group of hepatectomised rats at POD 10 compared to the control group. This difference was attributed to the higher neuronal metabolic rate during regeneration. The limitation of this study was that it was conducted at one time point after PH only (POD 10) thus restricting the significance of the findings [8].

The expression of GAP-43, a neuronal marker expressed in developing or regenerating axons, after PH is indicative of the presence of neural sprouting in the regenerating liver and indicates that the compensatory growth of the hepatic mass is the triggering stimulant. The timing of GAP-43 expression following PH is different than that of the liver cellular components or angiogenic events during the regenerative response. Hepatic mass and architecture are restored by POD 14 [3], whereas GAP-43 immnunoreactivty showed delayed onset at POD 5 and termination between 3 and 6 months post-PH. Ungvary et al. in 1974 were the first to study the effect of PH on liver innervation and concluded that nerve regeneration takes place in the regenerating organ, with the new neural elements growing out from intact nerves [7]. Neural sprouting was examined again in 1990, with the method of retrograde transport of horseradish peroxidase (HRP) for the study of vagal afferent neurons after PH in rats. At 3 weeks post-PH, no neural sprouting was evident in the regenerating liver [9].

The expression of PGP9.5 and GAP-43 was studied after OLT in rats and showed that hepatic reinnervation occurs in the transplanted liver at the porta hepatis [10,11]. The timing pattern of immunoreactivity has certain similarities to our study, with PGP9.5-positive axons present during the entire study period, whereas GAP-43-positive axons were present in some specimens at POD 3 and sustained until 3 months post-PH. No innervation was noted in the liver parenchyma [10,11]. The human transplanted liver, although inevitably denervated, maintains its function [22] and results from studies regarding hepatic reinnervation have been controversial. Both Dhillon et al. [23] and Boon et al. [24] have reported possible hepatic reinnervation after liver transplantation, while Kjaer et al. showed no evidence of liver sympathetic nerve fiber regeneration [25]. The latter finding is supported by a later study by May et al. who analysed sympathetic activation induced by water drinking on patients subjected either to OLT or to kidney transplantation and found reduced plasma norepinephrine levels in the OLT group after drinking water, implying impaired response to the stimulant due to liver graft denervation [26].

Hepatic nerve regeneration has been studied using GAP-43 immunohistochemistry in dogs following surgical denervation. GAP-43 positive nerve fibers were seen at 1 month post-denervation and absence of expression at 1 week and 3 months after the procedure was then observed [27].

Our study is the first to examine the expression of neuronal markers PGP9.5 and GAP-43 at several (n = 8) time points post-PH. Most of the related literature to date, with exception studies after OLT, originate from studies on liver innervation based on few time points only following either PH or surgical denervation. However, our study has certain limitations, namely the fact that it is based only in immunochemistry without the use of electron microscope and the porta hepatis was not examined. Furthermore, although many time points were used, it is difficult to reach to the general conclusion that the peak of hepatic reinnervation occurs at 3 months, since the interval until 6 months post-PH, when there is no expression of GAP-43, is quite long.

## Conclusion

Our results have shown that during liver regeneration after PH, the process of neural sprouting starts at POD 5 and terminates between 3 and 6 months post-PH. Taking into account the absence of intralobular innervation in the rat and the exclusive expression of both PGP9.5 and GAP-43 in the PTs, neural sprouting is considered to take place in the PTs of the regenerating liver. This finding indicates that possibly new PTs are created to accommodate the elongating nerve fibers. New PTs could further lead to new lobules as part of the regenerating response [28]. The question of new lobules or hyperplasia of existing ones during liver regeneration still remains and was addressed in the past by methods of measuring the size and number of liver lobules with conflicting results [29-33]. Further studies are needed to examine if GAP-43, a neuronal marker exclusively expressed in regenerating nerve axons and in PTs in the rat, could be used as a marker of new PTs in the regenerating rat liver, in order to accommodate the elongating nerve fibers, and further as a marker of new liver lobules.

## References

[1] Tiniakos DG, Kandilis A, Geller SA (2010). Tityus: a forgotten myth of liver regeneration. J Hepatol.

[2] Michalopoulos GK (2007). Liver regeneration. J Cell Physiol.

[3] Michalopoulos GK, DeFrances MC (1997). Liver regeneration. Science.

[4] Taub R (2004). Liver regeneration: from myth to mechanism. Nat Rev Mol Cell Biol.

[5] Fausto N, Campbell JS, Riehle KJ (2006). Liver regeneration. Hepatology.

[6] Martinez-Hernandez A, Amenta PS (1995). The extracellular matrix in hepatic regeneration. FASEB J.

[7] Ungvary G, Donath T, Naszaly SA (1974). Regeneration of the monoaminergic nerves in the liver after partial hepatectomy. Acta Morphol Acad Sci Hung.

[8] Pietroletti R, Chamuleau RA, Speranza V, Lygidakis NJ (1987). Immunocytochemical study of the hepatic innervation in the rat after partial hepatectomy. Histochem J.

[9] Carobi C (1990). Vagal afferent innervation in regenerated rat liver. Experientia.

[10] Sakamoto I, Takahashi T, Kakita A, Hayashi I, Majima M, Yamashina S (2002). Experimental study on hepatic reinnervation after orthotopic liver transplantation in rats. J Hepatol.

[11] Takahashi T, Kakita A, Sakamoto I, Takahashi Y, Hayashi K, Tadokoro F, Yamashina S (2001). Immunohistochemical and electron microscopic study of extrinsic hepatic reinnervation following orthotopic liver transplantation in rats. Liver.

[12] Wilkinson KD, Lee KM, Deshpande S, Duerksen-Hughes P, Boss JM, Pohl J (1989). **The neuron-specific protein PGP 9.5 is a ubiquitin carboxyl-terminal hydrolase**. Science.

[13] Wilson PO, Barber PC, Hamid QA, Power BF, Dhillon AP, Rode J, Day IN, Thompson RJ, Polak JM (1988). The immunolocalization of protein gene product 9.5 using rabbit polyclonal and mouse monoclonal antibodies. Br J Exp Pathol.

[14] Van Hooff CO, De Graan PN, Oestreicher AB, Gispen WH (1988). B-50 phosphorylation and polyphosphoinositide metabolism in nerve growth cone membranes. J Neurosci.

[15] Verge VM, Tetzlaff W, Richardson PM, Bisby MA (1990). Correlation between GAP43 and nerve growth factor receptors in rat sensory neurons. J Neurosci.

[16] Widmer F, Caroni P (1993). Phosphorylation-site mutagenesis of the growth-associated protein GAP-43 modulates its effects on cell spreading and morphology. J Cell Biol.

[17] Higgins GMAR (1931). Experimental pathology of the Liver. I. restoration of the liver of the white rat following partial surgical removal. Arch Pathol.

[18] Kogure K, Ishizaki M, Nemoto M, Kuwano H, Makuuchi M (1999). A comparative study of the anatomy of rat and human livers. J Hepatobiliary Pancreat Surg.

[19] Anagnostou VK, Doussis-Anagnostopoulou I, Tiniakos DG, Karandrea D, Agapitos E, Karakitsos P, Kittas C (2007). Ontogeny of intrinsic innervation in the human thymus and spleen. J Histochem Cytochem.

[20] Uyama N, Geerts A, Reynaert H (2004). Neural connections between the hypothalamus and the liver. Anat Rec A: Discov Mol Cell Evol Biol.

[21] Bioulac-Sage P, Lafon ME, Saric J, Balabaud C (1990). Nerves and perisinusoidal cells in human liver. J Hepatol.

[22] Colle I, Van Vlierberghe H, Troisi R, De Hemptinne B (2004). Transplanted liver: consequences of denervation for liver functions. Anat Rec A: Discov Mol Cell Evol Biol.

[23] Dhillon AP, Sankey EA, Wang JH, Wightman AK, Mathur S, Burroughs AK, Scheuer PJ (1992). Immunohistochemical studies on the innervation of human transplanted liver. J Pathol Bacteriol.

[24] Boon AP, Hubscher SG, Lee JA, Hines JE, Burt AD (1992). Hepatic reinnervation following orthotopic liver transplantation in man. J Pathol Bacteriol.

[25] Kjaer M, Jurlander J, Keiding S, Galbo H, Kirkegaard P, Hage E (1994). No reinnervation of hepatic sympathetic nerves after liver transplantation in human subjects. J Hepatol.

[26] May M, Gueler F, Barg-Hock H, Heiringhoff KH, Engeli S, Heusser K, Diedrich A, Brandt A, Strassburg CP, Tank J, Sweep FC, Jordan J: **Liver afferents contribute to water drinking-induced sympathetic activation in human subjects: a clinical trial.** *PLoS One* 2011, **6**(10):e25898. 10.1371/journal.pone.0025898 PMC3189227 22016786

[27] Ito Y, Takahashi T, Tadokoro F, Hayashi K, Iino Z, Sato K, Akira K (1998). Regeneration of the hepatic nerves following surgical denervation of the liver in dogs. Liver.

[28] Papp V, Dezso K, Laszlo V, Nagy P, Paku S (2009). Architectural changes during regenerative and ontogenic liver growth in the rat. Liver Transpl.

[29] Simpson GE, Finckh ES (1963). The pattern of regeneration of rat liver after repeated partial hepatectomies. J Pathol Bacteriol.

[30] Iashina IN (1970). Formation of hepatic lobules in the regenerating liver. Biull Eksp Biol Med.

[31] Sidorova VF (1959). On the structure of the regenerating liver in a rat. Biull Eksp Biol Med.

[32] Iatropoulos MJ (1971). Cytoarchitecture of rat liver during compensatory growth. Anat Rec.

[33] Wagenaar GT, Chamuleau RA, Pool CW, De Haan JG, Maas MA, Korfage HA, Lamers WH (1993). Distribution and activity of glutamine synthase and carbamoylphosphate synthase upon enlargement of the liver lobule by repeated partial hepatectomies. J Hepatol.

